# The spatial and temporal distribution of SARS-CoV-2 from the built environment of COVID-19 patient rooms: A multicentre prospective study

**DOI:** 10.1371/journal.pone.0282489

**Published:** 2023-03-13

**Authors:** Michael Fralick, Madison Burella, Aaron Hinz, Hebah S. Mejbel, David S. Guttman, Lydia Xing, Jason Moggridge, John Lapp, Alex Wong, Caroline Nott, Nicole Harris-Linton, Rees Kassen, Derek R. MacFadden

**Affiliations:** 1 Sinai Health System, Division of General Internal Medicine, Toronto, Ontario, Canada; 2 Lunenfeld-Tanenbaum Research Institute, Sinai Health System, Toronto, Ontario, Canada; 3 Sault Area Hospital, Sault Ste. Marie, Ontario, Canada; 4 Department of Biology, University of Ottawa, Ottawa, Ontario, Canada; 5 Department of Cell & Systems Biology, University of Toronto, Toronto, Ontario, Canada; 6 Centre for the Analysis of Genome Evolution & Function, University of Toronto, Toronto, Ontario, Canada; 7 The Ottawa Hospital Research Institute, Ottawa, Ontario, Canada; 8 Department of Biology, Carleton University, Ottawa, Ontario, Canada; VIT University, INDIA

## Abstract

**Background:**

SARS-CoV-2 can be detected from the built environment (e.g., floors), but it is unknown how the viral burden surrounding an infected patient changes over space and time. Characterizing these data can help advance our understanding and interpretation of surface swabs from the built environment.

**Methods:**

We conducted a prospective study at two hospitals in Ontario, Canada between January 19, 2022 and February 11, 2022. We performed serial floor sampling for SARS-CoV-2 in rooms of patients newly hospitalized with COVID-19 in the past 48 hours. We sampled the floor twice daily until the occupant moved to another room, was discharged, or 96 hours had elapsed. Floor sampling locations included 1 metre (m) from the hospital bed, 2 m from the hospital bed, and at the room’s threshold to the hallway (typically 3 to 5 m from the hospital bed). The samples were analyzed for the presence of SARS-CoV-2 using quantitative reverse transcriptase polymerase chain reaction (RT-qPCR). We calculated the sensitivity of detecting SARS-CoV-2 in a patient with COVID-19, and we evaluated how the percentage of positive swabs and the cycle threshold of the swabs changed over time. We also compared the cycle threshold between the two hospitals.

**Results:**

Over the 6-week study period we collected 164 floor swabs from the rooms of 13 patients. The overall percentage of swabs positive for SARS-CoV-2 was 93% and the median cycle threshold was 33.4 (interquartile range [IQR]: 30.8, 37.2). On day 0 of swabbing the percentage of swabs positive for SARS-CoV-2 was 88% and the median cycle threshold was 33.6 (IQR: 31.8, 38.2) compared to swabs performed on day 2 or later where the percentage of swabs positive for SARS-CoV-2 was 98% and the cycle threshold was 33.2 (IQR: 30.6, 35.6). We found that viral detection did not change with increasing time (since the first sample collection) over the sampling period, Odds Ratio (OR) 1.65 per day (95% CI 0.68, 4.02; p = 0.27). Similarly, viral detection did not change with increasing distance from the patient’s bed (1 m, 2 m, or 3 m), OR 0.85 per metre (95% CI 0.38, 1.88; p = 0.69). The cycle threshold was lower (i.e., more virus) in The Ottawa Hospital (median quantification cycle [Cq] 30.8) where floors were cleaned once daily compared to the Toronto hospital (median Cq 37.2) where floors were cleaned twice daily.

**Conclusions:**

We were able to detect SARS-CoV-2 on the floors in rooms of patients with COVID-19. The viral burden did not vary over time or by distance from the patient’s bed. These results suggest floor swabbing for the detection of SARS-CoV-2 in a built environment such as a hospital room is both accurate and robust to variation in sampling location and duration of occupancy.

## Introduction

SARS-CoV-2 primarily spreads via aerosols and droplets, and the degree of aerosolization is related to multiple factors, including ventilation [1–4]. Within the built environment, the floor is the most common location where the virus can be detected [5–9]. Floors likely act as a “sink,” collecting the droplets and aerosols produced by infected individuals when those particles eventually fall to the floor. Our previous research was one of the first studies to identify whether the SARS-CoV-2 virus can be detected from the built environment within a hospital [5]. We conducted a multicentre prospective study at two hospitals in Ottawa, Ontario, Canada in which high touch surfaces (e.g., computer keyboard, door handle, telephone receiver, various equipment) and the floors were swabbed weekly for a total of ten weeks. We were able to recover viral ribonucleic acid (RNA) from these surfaces on wards dedicated to patients with COVID-19, but not on wards where there were no patients with COVID-19. The floor was the most common surface where the virus was detected, and this observation has been replicated in other studies [5, 6, 8]. A limitation of this study was that we did not swab within patient rooms, and instead swabbed only the hallways of wards and other common areas within the hospital.

One of the first studies swabbing inside the rooms of patients with COVID-19 was by Zhang et al. [8]. They collected over 2000 environmental swabs on inpatient wards, including in common areas and in the rooms of patients with COVID-19. The percentage of swabs positive for SARS-COV-2 in the common areas was 75%, and within the patient rooms was slightly higher (77%) [8]. Kim et al. conducted a study at four hospitals in Korea assessing both air and surface contamination, as well as the impact of surface cleaning on the ability to detect SARS-CoV-2 [10]. They collected 330 swabs, of which 27% were positive for SARS-CoV-2 [10]. Following routine cleaning procedures, they were unable to detect the virus on the surfaces. Taken together, these data suggest that environmental sampling could be a method of non-invasive surveillance for COVID-19.

Because the studies by Zhang et al. and Kim et al. did not conduct serial swabs surrounding the patient, it is unknown how the viral burden surrounding a patient changes over space and time [8, 10]. One single-centre study in the United States swabbed within the rooms of patients with COVID-19 to determine how severity of illness and distance from the patient’s bed affected the recovery of SARS-CoV-2 from floors and high touch surfaces [11]. The researchers included 111 unique patient-room pairs and conducted a median of 1.5 swabs per patient-room pair. The probability of detecting SARS-CoV-2 from the floor was approximately 80% and did not vary over distance, but was higher for patients with more severe disease (e.g., requiring positive pressure ventilation) [11]. Because they did not perform serial swabs each day within the patient’s room, it is unknown how the probability of detection would vary over time. Furthermore, because their study was single-centre and observational, they were unable to identify how different cleaning protocols affected their findings. Our objective was to conduct serial swabs at systematic distances and times to understand how the viral burden changes over space and time, and how different cleaning protocols affect viral burden.

## Methods

### Study design

We conducted a multicentre prospective study at two tertiary care hospitals in Ontario, Canada (Mount Sinai Hospital in Toronto and The Ottawa Hospital in Ottawa) between January 19, 2022 and February 11, 2022. Both hospitals have a combination of single and multi-patient rooms; however, we only included single rooms, where patients were hospitalized for COVID-19 in the preceding 48 hours. These rooms represented a convenience sample of patient rooms that met the study inclusion criteria. We swabbed the floors twice daily (at 9:00 and 17:00), at three distances from the hospital bed: 1 metre (m), 2 m, and where the room connected with the hallway (typically 3 to 5 m from the patient’s bed). Patient rooms were fully cleaned and disinfected before and after each admission. At The Ottawa Hospital, the floors and bathrooms of patient rooms were cleaned once daily while occupied by a SARS-CoV-2-infected patient; the floors and bathrooms at Mount Sinai Hospital were cleaned twice daily. Patient consent was not required because our study did not collect any patient-level data, and we were sampling floors, not human subjects. At The Ottawa Hospital, this study was conducted under an existing Research Ethics Board approval, and at Mount Sinai Hospital the research was deemed excluded from requiring institutional approval, given that it does not involve direct work with human subjects.

### Environmental detection of SARS-CoV-2 by quantitative reverse transcriptase polymerase chain reaction (RT-qPCR)

Trained research staff swabbed the floors, with each sample involving approximately 30 seconds of swabbing across a 2” x 2” area. Floors were sampled using the P-208 Environmental Surface Collection Prototype kit from DNA Genotek (provided in-kind). The kit includes a flocked swab and 2 mL of semi-lytic nucleic acid stabilization solution for post-collection swab immersion. SARS-CoV-2 was detected by quantitative reverse transcriptase polymerase chain reaction (RT-qPCR) of the viral N-gene from RNA extracted from the stabilization solution using the MagMAX Viral/Pathogen II (MVP II) Nucleic Acid Isolation Kit (Thermo Fisher Scientific, Waltham, MA). Our previous study provides in-depth information on the validation of SARS-CoV-2 detection from built environment swabs [5]. The validation study included identifying the limit of detection and quantification using 10-fold serial dilutions of SARS-CoV-2, in addition to validating the swabbing method in laboratory experiments where various materials were spiked with inactivated SARS-CoV-2. The RT-qPCR results provided us with a quantification cycle (Cq) of detection for each positive swab; we estimated the number of viral copies present using the Cq values and a previously determined standard curve [5]. For this study, we considered a positive result to be a Cq less than 45, which is an accepted cut-off used for environmental surveillance of SARS-CoV-2 [12].

### Study outcomes

We hypothesized that SARS-CoV-2 detection would decrease with increasing distance from the patient’s bed and decrease over time from admission. We quantified the percentage of floor swabs positive for SARS-CoV-2, as well as the number of viral copies recovered per positive swab, and how these changed over space (e.g., distance from the patient’s bed) and time in each room.

### Statistical analysis

All statistical analyses were performed using the R language (v4.1.2) [13], and all figures were created with the ‘ggplot2’ package (v3.3.6). We used descriptive statistics to compare swab results (e.g., positivity and number of viral copies) over space and time. We calculated the sensitivity of surface swabbing under the assumption that all swabs would detect SARS-CoV-2 in the area immediately surrounding the patient. Confidence intervals (CI) for sensitivity estimates were computed using the Agresti-Coull method for binomial proportions (using the `binom`package v1.1). Seventeen samples were lost or spoiled after collection and could not be tested; these observations were treated as missing at random in our analyses.

To examine differences in SARS-CoV-2 detection between hospitals, we first computed the room-level means for the proportion of positive swabs. Similarly, we computed the room-level (geometric) means for the number of viral copies using the log_10_ transformed values to reduce positive skew. We performed two-tailed student’s t-tests to compare hospitals using the room-level means for each outcome to avoid pseudoreplication.

We examined the effects of time and distance on the detection of SARS-CoV-2 using hierarchical mixed-effects models. In each model, random intercepts were included to account for correlation in the data due to repeated observations within rooms and the clustering of rooms within hospitals. A mixed-effects logistic regression model was created with SARS-CoV-2 detection as a binomial outcome with logit link function, where model parameters were estimated by maximum-likelihood using the Laplace approximation and Nelder-Mead optimization (using `glmer`from ‘lme4’ v1.1). For fixed effects, we estimated odds ratios and 95% CI (Wald score method). Model fit was assessed by examining the residuals, fitted values, and dispersion. We left the values for time or distance unstandardized, such that their effect sizes could be interpreted in terms of days or metres, respectively. We used the unstructured default variance-covariance matrix for lme4.

We used the number of viral copies as a numeric outcome for a linear mixed-effects model to examine the effects of time and distance on the quantity of SARS-CoV-2 recovered from positive surfaces. Random intercepts were included for rooms clustered within hospitals. This model was created using the `lmer`function from the ‘lme4’, with restricted maximum likelihood estimation and an unstructured covariance matrix. Data are available for sharing; researchers can visit https://cube-ontario.github.io/.

## Results

### Overall findings

**Fig 1:**
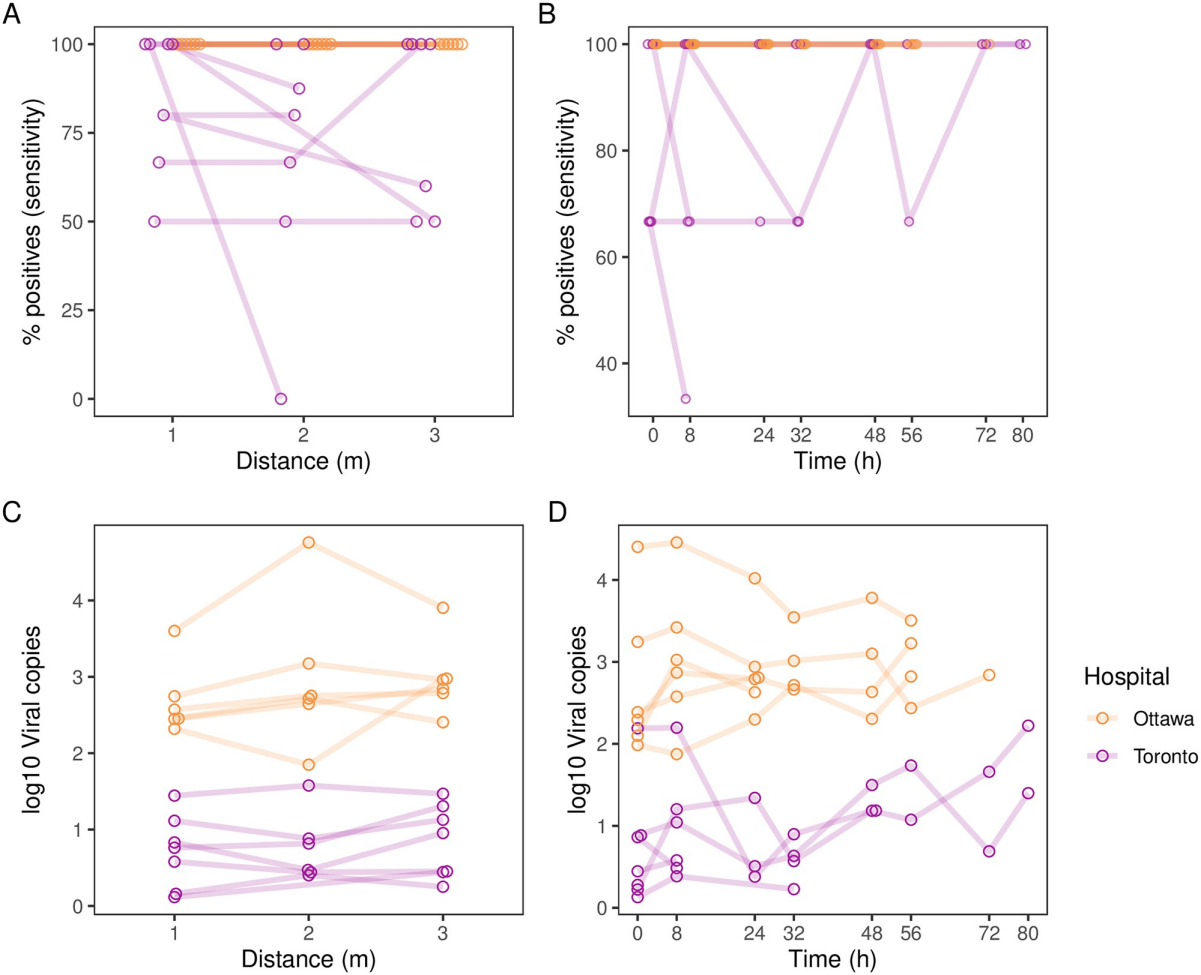
Detection of SARS-CoV-2 (positivity and viral copies) in COVID-19 patient rooms over (A & C) space and (B & D) time. m = metre, h = hour. Distance was measured from the patient’s bed, with 3 m indicating where the room connected with the hallway (typically 3 m to 5 m from the patient’s bed). Time 0 is the first swab sample taken.

**Fig 2:**
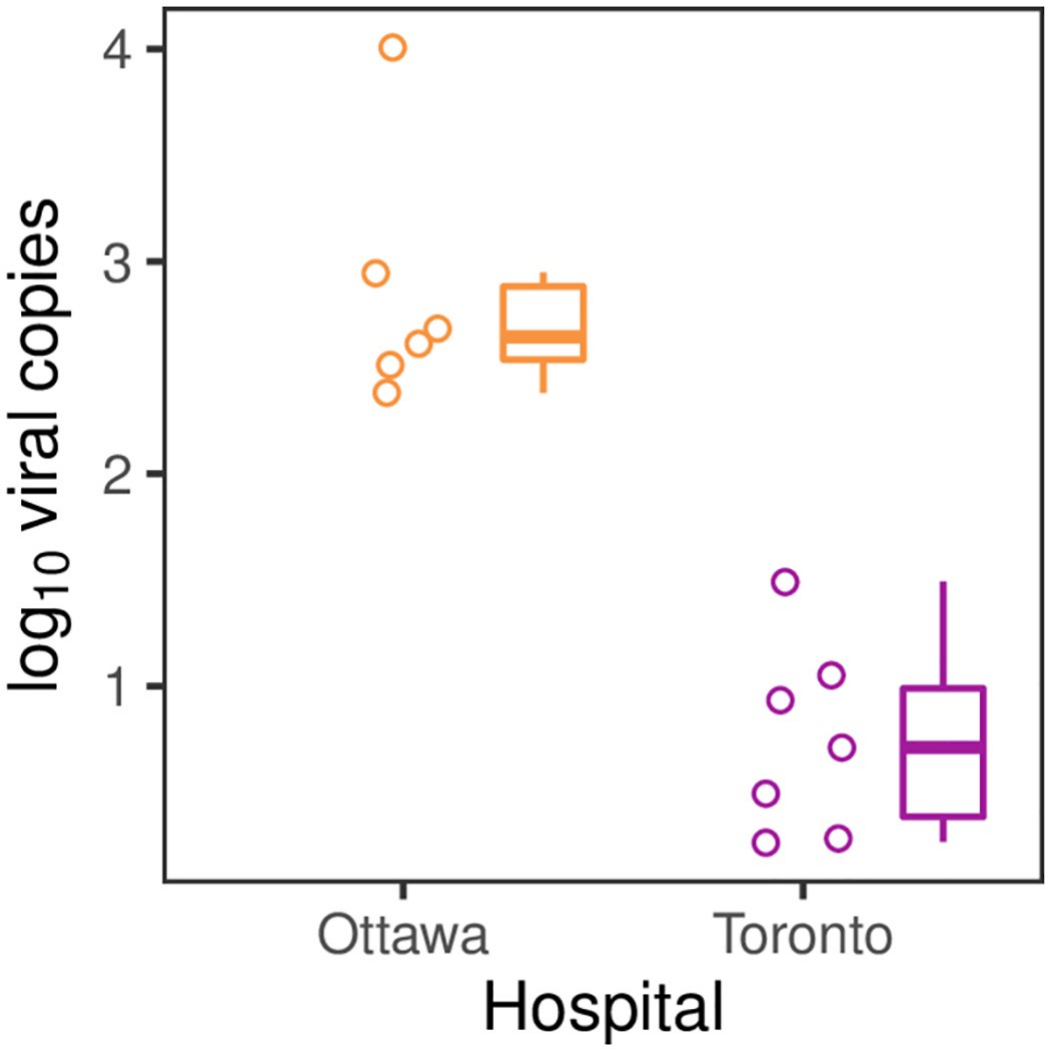
A comparison of detected SARS-CoV-2 quantities across COVID-19 patient rooms at two hospitals. Points show the patient room means of log_10_ viral copies from all the positive swabs collected (negative results were excluded from the calculation of room-level means). Boxplots summarize these values for each hospital.

**Fig 3:**
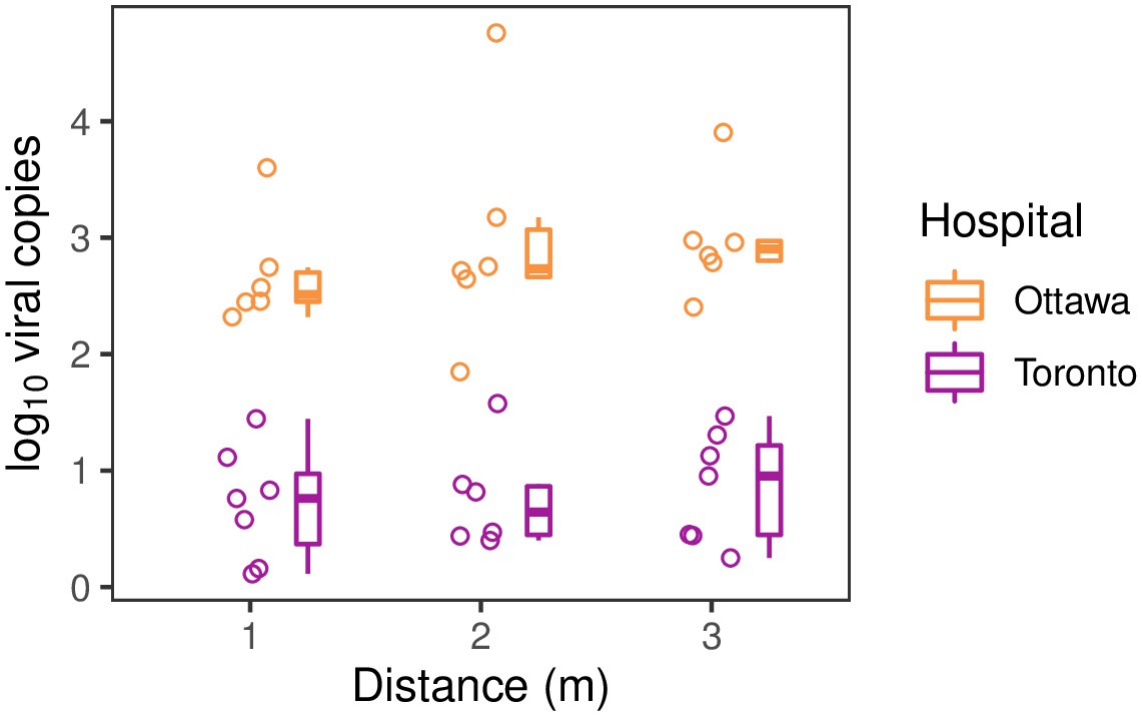
A comparison of detected SARS-COV-2 at varying distances across COVID-19 patient rooms at two hospitals. m = metre. Distance was measured from the patient’s bed, with 3 m indicating where the room connected with the hallway (typically 3 m to 5 m from the patient’s bed). Points show the patient room means of log_10_ viral copies from all the positive swabs collected (negative results were excluded from the calculation of room-level means). Boxplots summarize these values for each hospital.

Over the 6-week study period, we collected 164 floor swabs from the rooms of 13 patients newly hospitalized with COVID-19 (Table 1). The overall percentage of swabs positive for SARS-CoV-2 was 93% and the median cycle threshold was 33.4 (interquartile range [IQR]: 30.8, 37.2). Overall, the median patient-room observation period lasted 48 hours (h). However, patients tended to drop out earlier at Mount Sinai Hospital (Toronto), with a median observation period of 32 h, compared to 55 h at The Ottawa Hospital (Ottawa). Rooms where patients stayed longer generally had slightly greater SARS-CoV-2 detection in terms of sensitivity (0.09 ± 0.03 per day, F = 2.7, p = 0.02), but the number of copies recovered did not change significantly with the duration of the patient’s stay (0.42 ± 0.29 per day, F = 1.5, p = 0.17) (Figs 1–3).

**Table 1:** Environmental detection of SARS-CoV-2 RNA in patient rooms.

	Patient rooms (n)	Samples (n)	Positives (n)	Mean Sensitivity (95% CI)	Median (IQR) log10 Copies
**Overall**					
	13	164	152	0.93 (0.88, 0.96)	2.08 (0.9, 2.88)
**Day 0**					
Overall	13	66	58	0.88 (0.78, 0.94)	2.02 (0.6, 2.58)
1 m	13	23	21	0.91 (0.72, 0.99)	2 (0.76, 2.43)
2 m	13	24	21	0.88 (0.68, 0.96)	2.05 (0.44, 2.68)
3 m	11	19	16	0.84 (0.62, 0.95)	1.98 (0.73, 2.3)
**Day 1**					
Overall	10	46	43	0.93 (0.82, 0.98)	2.32 (0.79, 2.97)
1 m	10	17	16	0.94 (0.71, 1)	2.57 (0.83, 2.96)
2 m	8	14	13	0.93 (0.66, 1)	1.21 (0.76, 3.11)
3 m	9	15	14	0.93 (0.68, 1)	2.58 (0.82, 2.79)
**Day 2**					
Overall	7	35	34	0.97 (0.84, 1)	2.27 (1.48, 3.09)
1 m	7	13	13	1 (0.73, 1)	2.25 (1.53, 2.57)
2 m	7	10	9	0.9 (0.57, 1)	2.22 (1.46, 3.1)
3 m	7	12	12	1 (0.72, 1)	2.52 (1.52, 3.66)
**Day 3**					
Overall	3	17	17	1 (0.78, 1)	1.53 (1.03, 2.37)
1 m	3	5	5	1 (0.51, 1)	1.36 (1.03, 1.54)
2 m	3	5	5	1 (0.51, 1)	2.21 (1.52, 2.42)
3 m	3	7	7	1 (0.6, 1)	1.53 (0.78, 2.38)

CI = confidence interval, IQR = interquartile range, m = metre. Distance was measured from the patient’s bed, with 3 m indicating where the room connected with the hallway (typically 3 m to 5 m from the patient’s bed). Day 0 is the first day swab samples were taken.

### SARS-CoV-2 viral detection over time and distance

We created a mixed-effects logistic regression model to evaluate the effects of time and distance on SARS-CoV-2 viral detection in patient rooms, with random intercepts specified for rooms clustered within hospitals. We found that viral detection did not change with increasing time (since the first sample collection) over the sampling period, Odds Ratio (OR) 1.65 per day (95% CI 0.68, 4.02; p = 0.27). Similarly, viral detection did not change with increasing distance from the patient’s bed (1 m, 2 m, or 3 m), OR 0.85 per metre (95% CI 0.38, 1.88; p = 0.69). The variance of the fixed effects time and distance (0.27) was very small compared to the variance of the random effects associated with hospital and room (7.69), while the residual variance was 3.29. The variance of the random intercepts for rooms (0.73; 9.5% of random effects variance) was very small compared to the variance for hospitals (6.96; 90.5%). A linear mixed model with the number of viral copies recovered as a continuous outcome showed similarly null results for the effects of time (estimate 0.0572; 95% CI -0.045, 0.16) and distance (estimate 0.10; 95% CI -0.01, 0.22), with random effects accounting for the majority of variance.

### Comparison between hospitals

We observed large differences in environmental SARS-CoV-2 detection in patient rooms between the two hospitals. At The Ottawa Hospital (Ottawa), 100% of samples were positive for SARS-CoV-2 for all rooms, whereas at Mount Sinai Hospital (Toronto) the mean proportion of positives per room was only 78% (95% CI: 62–94%; p < 0.05). The mean number of viral copies quantified in positive samples was much greater for The Ottawa Hospital (719 copies; 95% CI: 171–3,017 copies) than Mount Sinai Hospital (5.6 copies; 95% CI: 2.1–14 copies; p < 0.0001).

## Discussion

In this multicenter prospective study, we were able to identify and recover SARS-CoV-2 from the floors surrounding patients with COVID-19 in all of the included rooms. The viral burden did not increase over time, and the virus was consistently identified at 1 m and 2 m from the hospital bed, as well as at the entryway to the room. The calculated sensitivity for swabbing the floor was 93%, indicating that floors serve as accurate indicators of the presence of patients with COVID-19.

Our findings align with the body of literature that SARS-CoV-2 can be detected from the floors in areas where there are patients with COVID-19. In our study, 100% of patients had at least one swab positive for SARS-CoV-2 on the first day of swabbing, which demonstrates that accumulation of viral particles on the floors occurs quickly. Patients were generally unmasked, so most expelled viral particles presumably end up on the floor. Our findings also suggest that frequent cleaning procedures may result in a lower burden of virus recovered, as SARS-CoV-2 RNA was detected more frequently at the hospital with once daily cleaning than at the hospital with twice daily cleaning. We did not evaluate whether swabbing immediately after cleaning resulted in an inability to detect SARS-COV-2; however, prior studies have done so [10, 14, 15]. In the study by Kim et al., RNA was not detected in a room routinely cleaned by disinfectant wipes, demonstrating how cleaning removes SARS-CoV-2 from the surface; however, RNA was detected in a room sprayed with disinfectant, suggesting disinfectant sprays may not be effective in reducing exposure [10]. These findings confirm that what is detected on the floor is not simply a reflection of prior patients in the room.

We also identified that the virus could be consistently identified at all distances from the patient’s bed where we swabbed (i.e., 1 m, 2 m, entryway to the room). It is important to note that the rooms we studied had patients who are typically confined to their beds because of their oxygen requirements from the severe fatigue and weakness of COVID-19. Thus, our ability to consistently detect the virus at increasing distances from the patient’s bed goes against the historically referenced “Six-Foot Rule” [16]. The Six-Foot Rule—which states that staying 6 feet apart could help prevent the spread of COVID-19—was recommended by the Centers for Disease Control and Prevention and other agencies based on the assumption that COVID-19 is spread via large droplets that can only travel short distances [17]. Our study adds to the available literature that the virus can rapidly reach distances beyond 6 feet.

Our results highlight that swabbing floors for SARS-CoV-2 may be a practical tool for viral surveillance in settings where individual testing is not regularly performed. This environmental sampling technique may help identify locations of outbreaks, predict future outbreaks in advance of confirmed cases, and guide disinfection protocols in healthcare settings [5].

There are important limitations to our study. First, while we conducted 164 swabs, our study only included the rooms of 13 unique patients. As a result, our null findings that the viral detection did not change with increasing time or distance may reflect a lack of statistical power. For that reason, we also calculated the 95% CI to capture the uncertainty surrounding our point estimates (i.e., odds ratios). Second, we did not collect patient-level data to investigate how severity of illness or the degree to which a patient was immunocompromised may have influenced the viral burden detected. However, a prior study observed higher rates of surface contamination with SARS-CoV-2 for patients who required high-flow oxygen or positive pressure ventilation compared to hospitalized patients with less severe illness (OR = 1.6, 95% credible interval [CrI] 1.03–1.25) [11]. Third, by definition we only included rooms of patients with COVID-19 who were hospitalized on a medical ward, and thus it is unknown whether our findings apply for patients with mild disease who did not require hospitalization. Finally, our study focused on SARS-CoV-2; it is unknown how our results will apply to other respiratory pathogens, such as influenza or respiratory syncytial virus. This will be an important area of future work, for which our study design can be easily adapted.
